# Mathematical pattern of Kessler psychological distress distribution in the general population of the U.S. and Japan

**DOI:** 10.1186/s12888-021-03198-y

**Published:** 2021-04-10

**Authors:** Shinichiro Tomitaka, Toshiaki A. Furukawa

**Affiliations:** 1 Department of Health Promotion and Human Behavior, Kyoto University Graduate School of Medicine/School of Public Health , Yoshida Konoe-cho, Sakyo-ku, Kyoto, 606-8501 Japan; 2 Department of Clinical Epidemiology, Kyoto University Graduate School of Medicine/School of Public Health, Yoshida Konoe-cho, Sakyo-ku, Kyoto, 606-8501 Japan; 3 Department of Mental Health, Panasonic Health Center, Landic building 3F, Nishishinbashi 3-8-3, Minato-ku, Tokyo, 105-0003 Japan

**Keywords:** Psychological distress, Depressive symptom, Mathematical model, *Exponential distribution*, National Health Interview Survey, National Survey on drug use and health, Behavioral risk factor surveillance system, Comprehensive survey of living conditions

## Abstract

**Background:**

Although the 6-item Kessler psychological scale (K6) is a useful depression screening scale in clinical settings and epidemiological surveys, little is known about the distribution model of the K6 score in the general population. Using four major national survey datasets from the United States and Japan, we explored the mathematical pattern of the K6 distributions in the general population.

**Methods:**

We analyzed four datasets from the National Health Interview Survey, the National Survey on Drug Use and Health, and the Behavioral Risk Factor Surveillance System in the United States, and the Comprehensive Survey of Living Conditions in Japan. We compared the goodness of fit between three models: exponential, power law, and quadratic function models. Graphical and regression analyses were employed to investigate the mathematical patterns of the K6 distributions.

**Results:**

The exponential function had the best fit among the three models. The K6 distributions exhibited an exponential pattern, except for the lower end of the distribution across the four surveys. The rate parameter of the K6 distributions was similar across all surveys.

**Conclusions:**

Our results suggest that, regardless of different sample populations and methodologies, the K6 scores exhibit a common mathematical distribution in the general population. Our findings will contribute to the development of the distribution model for such a depression screening scale.

**Supplementary Information:**

The online version contains supplementary material available at 10.1186/s12888-021-03198-y.

## Background

The 6-item Kessler psychological scale (K6) is a useful instrument to screen for depressive and anxiety disorders, and it is often used in national surveys for the assessment of psychological distress worldwide [1]. In the United States, the K6 has been used for major national health surveys, such as the National Health Interview Survey (NHIS), the National Survey on Drug Use and Health (NSDUH) and the Behavioral Risk Factor Surveillance System (BRFSS) [2]. To date, using such data, a considerable amount of literature has been published on the psychometric properties of the K6, such its internal consistency, reliability, construct validity, and item response characteristics [3, 4]. However, little attention has been paid to the pattern of the K6 distributions in the general population.

While analyzing data using such scales, researchers often employ statistical methods that assume a normal distribution, such as parametric statistics, factor analysis, and Cronbach’s alpha coefficient [5]. A noteworthy fact is that in such epidemiological surveys, normality is rarely observed in the K6 distribution [6, 7]. Because the majority of individuals in a general population have a little or no psychological distress, the K6 score distributions are usually right-skewed [7]. However, few researchers have attempted to elucidate the mathematical patterns of the skewed distributions.

Recent studies have revealed that total scores on the K6, the Center for Epidemiologic Studies Depression Scale (CES-D), the nine-item Patient Health Questionnaire (PHQ-9), and the Revised Clinical Interview Schedule (CIS-R) exhibit an exponential pattern, except for the lower end of the distribution in the general population [7–10]. Melzer et al. were the first to report that an exponential curve provided the best fit for the CIS-R distribution from a wide range of potential statistical distributions, including exponential, Pareto, log-normal, Weibull, and Gamma curves [10]. We also confirmed that an exponential curve provided the best fit for the total score distribution on the CES-D, PHQ-9, and K6 [11]. Furthermore, in our previous studies, we analyzed the summed scores of 2 items, 3 items, 4 items, and 5 items on the K6 in various combinations and found that for any combination, if the number of chosen items was same, the summed scores approximated an exponential pattern with a similar rate parameter, except at the lowest scores [12]. Equivalent results were obtained when we analyzed the distributions of the summed scores of the chosen items on the CES-D in various combinations [13]. Generally, the results of item responses on such scales differ depending on sample population and survey design. However, based on the results so far, regardless of the different sample populations and survey designs, the total score distributions on this kind of a depression screening scale may follow the same mathematical distribution in the general population, that is an exponential pattern with a similar rate parameter except at the lowest scores.

The K6 is used in several national health surveys in the United States and Japan. The demographics of the United States differ from those of Japan in terms of age distribution and diversity [14]. In the present study, analyzing four representative datasets from American and Japanese national surveys using the K6, we compared the goodness of fit between the exponential, power law, and quadratic function models. Thereafter, we investigated whether the K6 total distributions exhibit an exponential pattern with a similar rate parameter regardless of the different sample populations and survey methods.

## Methods

### Dataset

This study used four datasets: the 2018 NHIS, the 2014 NSDUH, and the 2013 BRFSS in the United States, as well as the 2016 Comprehensive Survey of Living Conditions (CSLC) in Japan. The NHIS, NSDUH, and BRFSS are the major surveys for mental health indicators in the United States mainly sponsored by the National Center for Health Statistics, the Substance Abuse and Mental Health Services Administration, and the Centers for Disease Control and Prevention, respectively. The 2018 NHIS, 2014 NSDUH, 2013 BRFSS, and 2016 CSLC were used because they are the latest available K6 datasets from each survey: the BRFSS has not included the K6 after the 2013 BRFSS and the CSLC had not included the K6 in 2017 and 2018.

NHIS is a continuous nationally representative sample survey that collects data on a broad range of health measures using personal house interviews [15]. NSDUH is the primary source of the use of illegal drugs by the US population and also includes indicators of mental health problems [16]. BRFSS is a state-based system of health surveys that collect information on health-risk behaviors, clinical preventive practices, and health care access [17]. CSLC is a repeated national cross-sectional survey that evaluates the basic living conditions of residents in Japan [18]. NHIS, NSDUH, and BRFSS datasets are accessible to researchers worldwide through their official repositories. The CSLC dataset is accessible to researchers upon approval by the Ministry of Health, Labour and Welfare of Japan through specific application procedures.

The NHIS is a multistage probability sample survey of the civilian, noninstitutionalized US population aged ≥18 years. The NHIS sample included 25,417 respondents, and the total household response rate was 64.2%. The NSDUH uses a national probability sample of the US civilian, noninstitutionalized population aged ≥12 years (18 years or older for this analysis). The NSDUH sample included 55,272 respondents, with a response rate of 71.2%.

The BRFSS is a population-based state surveillance system that uses random-digit-dialed telephone surveys of noninstitutionalized US citizens aged ≥18 years. The BRFSS questionnaire includes optional modules on specific topics. In 2013, four states: Minnesota, Nevada, Tennessee, and Washington used an optional module of Mental Illness and Stigma that included the K6. The total 2013 K6 sample comprised 33,211 respondents, including 12,781, 4564, 5611, and 10,255 respondents for Minnesota, Nevada, Tennessee, and Washington, with response rates of 54.3, 43.7, 45.9, and 31.1%, respectively.

The survey unit of CSLC includes households and household members; they are randomly selected by stratified random sampling method of census enumeration districts in Japan, covering approximately 300,000 households. The response rate of the survey was 77.5%. This study used CSLC data from a total of 220,294 participants (≥18 years old). Thus, all the participants for the four datasets were adults aged ≥18 years. The socio-demographic characteristics of the participants of the four surveys have been reported in detail elsewhere [19–22].

### Ethics statement

This study is a secondary analysis of freely accessible public data. The ethics committees of Kyoto University Graduate School of Medicine and Panasonic Health Center do not consider de-identified public data analysis to be human research and, thus, the need for ethical approval was waived.

### Measures

The standard K6 includes six items related to the degree with which participants have felt (1) nervous, (2) hopeless, (3) restless or fidgety, (4) so depressed that nothing could cheer them up, (5) that everything was an effort, and (6) worthless over the previous 30 days [1]. Each item was self-rated on a 5-point scale ranging from 0 = “none of the time” to 4 = “all of the time,” yielding a total item score range of 0–24. Respondents with a total score of 13 or greater are classified as having past month serious psychological distress (SPD). Although all surveys use the K6 to assess past month psychological distress and the same cutoff score for SPD, variations exist in the wording of question items among the surveys. For example, the fourth K6 item used in the NHIS survey is worded as “*feel so sad,*” while the one in the NSDUH is “*feel so sad or depressed*” and the one in the BRFSS is “*feel so depressed*.” The CSLC uses the Japanese version of the K6, which is developed in accordance with the World Health Organization (WHO) translation guidelines and shown to have screening performances equivalent to that of the original English version [23].

### Analysis

First, we calculated the descriptive statistics of the K6 score distributions for the NHIS, NSDUH, BRFSS, and CSLC. As our hypothesis was not based on equality of distributions, but on similarity of distribution patterns regardless of regardless of different sample populations and methodologies, we did not estimate significant differences based on the null hypothesis that the mean values and PSD percentages were equal.

Next, we compared the goodness of fit between the three models: exponential, power law, and quadratic function models. The Corrected Akaike’s Information Criterion (AICc), Schwarz’s Bayesian Information Criterion (BIC), and Root Mean Square Error (RMSE) were calculated to compare the fits. With regard to AICc and BIC, models with smaller values are considered to be a better fit. A fitting curve was calculated using the method of least squares.

Moreover, we graphically analyzed the patterns of the K6 total score distributions from the four surveys using normal and logarithmic scales. Although histograms are generally used for ordinal variables, we used line charts representing the K6 total score distributions because we cannot plot multiple histograms together on a single graph. Thus, we used line charts to show the common mathematical pattern across the four surveys. All statistical procedures were performed using JMP Version 15.0.0 for Windows (SAS Institute, Inc., Cary, NC, US). Because the purpose of the present research was to investigate a mathematical pattern of sampling distribution, all analyses were based on raw data.

## Results

### Sample characteristics

This present analysis used data from participants aged ≥18 years, excluding those who did not respond to all items. The final sample for the analysis included 24,683, 41,082, 31,502, and 201,116 respondents for the NHIS, NSDUH, BRFSS, and CSLC, respectively. Demographic characteristics of the final sample for the four surveys are summarized in Table 1.

**Table 1:** Demographic characteristics of the four survey samples

	2018 NHIS (*n* = 24,683)	2014 NSDUH (*n* = 41,082)	2013 BRFSS (*n* = 31,503)	2016 CSLC (*n* = 201,116)
**Sex**				
Male	11,223 (45.5%)	19,141 (46.6%)	13,042 (41.4%)	96,036 (47.8%)
Female	13,460 (54.5%)	21,941 (53.4%)	18,460 (58.6%)	105,080 (52.2%)
**Age**				
18–34	5582 (22.6%)	21,100 (51.4%)	4400 (14.0%)	21,262 (10.6%)
35–49	5615 (22.7%)	11,099 (27.0%)	6546 (20.8%)	43,822 (21.8%)
50–64	6395 (25.9%)	5317 (12.9%)	10,645 (33.8%)	52,334 (26.0%)
≧65	7091 (28.7%)	3566 (8.7%)	10,001 (31.7%)	83,698 (41.6%)
**Race**^a^				
White	19,644 (79.6%)	25,788 (62.8%)	27,136 (86.1%)	–
Black	2862 (11.6%)	4822 (11.7%)	1358 (4.3%)	–
Asian	1288 (5.2%)	1783 (4.3%)	568 (1.8%)	–
AI/AN	284 (1.2%)	668 (1.6%)	297 (0.9%)	
Hispanic	3106 (12.6%)	6572 (16.0%)	1284 (4.1%)	–
Other races	605 (2.5%)	1449 (3.5%)	859 (2.7%)	–

American Indian/Alaskan Native (AI/AN). CSLC has not asked about ethnicity. ^a^The method of measuring race and ethnicity differs depending on the survey. Especially, the CSLC did not measure race and ethnicity

As shown in Table 1, the age distribution differs among the three surveys. Specifically, the NSDUH had an overwhelmingly larger number of participants aged between 18 and 35 years than did the other surveys. Conversely, the CSLC had a smaller number of participants aged between 18 and 35 years than did the other surveys. Moreover, the CSLC did not measure race and ethnicity. According to Japanese government statistics, the proportion of Asians in Japan is estimated to exceed 98% [24].

### Descriptive statistics of the K6 distributions among the national surveys

The descriptive statistics of the K6 score distributions for the NHIS, NSDUH, BRFSS, and CSLC are shown in Table 2.

**Table 2:** Descriptive statistics for the K6 distributions of the four major surveys

Survey	N	Mean ± S.D.	Skewness	Kurtosis	Median	SPD
**NHIS**	24,683	2.9 ± 4.1	2.0	4.7	1	4.0%
**NSDUH**	41,082	4.3 ± 4.5	1.5	2.4	3	6.4%
**BRFSS**	31,502	2.8 ± 3.8	2.2	5.8	2	3.6%
**CSLC**	201,116	2.8 ± 3.6	1.7	2.8	1	3.9%

*N* number of samples, *SD* standard deviation, *SPD* the prevalence of serious psychological distress (a score of ≧13 on K6) All descriptive statistics were based on raw data

The mean value of the K6 total score was apparently higher in the NSDUH (4.3) than in the NHIS (2.9), BRFSS (2.8), and CSLC (2.8). The skewness values for the NHIS (2.0), NSDUH (1.5), BRFSS (2.2), and CSLC (1.7) were close to 2. Mathematically, the skewness of any exponential distributions is 2. Consistent with the findings of the mean value of the K6 total scores, the prevalence of SPD (a score of ≧13 on K6) was higher in the NSDUH (6.4%) than in the NHIS (4.0%), BRFSS (3.6%), and CSLC (3.9%).

### Goodness of fit of the three models

Table 3 summarizes the results of the goodness of fit of the three models. The exponential model showed smaller BIC and AICc values than did the quadratic or power law models for the four surveys, indicating that the exponential curve had the best fit. The coefficients of determination ranged from 0.94 to 0.97, indicating that the R-squared values are greater than those for regression with quadratic term (0.60–0.91) and power law (0.82–0.93) functions. The rate parameter of the fitting exponential function for all four surveys ranged from − 0.21 to − 0.23, indicating a similar parameter across all surveys.

**Table 3:** Comparison of the fitting of the three models

Model	Data	AICc	BIC	RMSE	R^**2**^	Fitting curve
**Exponential**	NHIS	− 109.9	− 107.4	0.02	0.96	y = 0.1945e^-0.22x^
	NSDUH	− 151.8	− 149.3	0.01	0.96	y = 0.2302e^-0.21x^
	BRFSS	− 145.2	− 142.7	0.01	0.97	y = 0.2057e^-0.23x^
	CSLC	−106.8	− 104.3	0.03	0.94	y = 0.2193e^-0.23x^
**Quadratic**	NHIS	−69.5	−66.6	0.05	0.64	y = 0.0009 × ^2^–0.03x + 0.23
	NSDUH	− 128.7	− 125.9	0.02	0.91	y = 0.0005 × ^2^–0.02x + 0.18
	BRFSS	−88.0	−85.2	0.04	0.78	y = 0.0009 × ^2^–0.03x + 0.23
	CSLC	−68.6	−65.8	0.05	0.60	y = 0.0008 × ^2^–0.03x + 0.22
**Power law**	NHIS	− 89.2	− 86.9	0.04	0.91	y = 0.8969x^-1.90^
	NSDUH	−98.3	− 96.2	0.03	0.82	y = 0.7778x^-1.68^
	BRFSS	− 120.3	− 118.9	0.06	0.93	y = 1.0261x^-1.98^
	CSLC	−82.3	−80.3	0.04	0.85	y = 0.945x^-1.91^

*AICc* Corrected Akaike’s Information Criterion, *BIC* Schwarz’s Bayesian Information Criterion and. *RMSE* Root Mean Square Error. R^2^ is the coefficient of determination

The independent variable, x, is the K6 total score and the dependent variable, y, is the relative frequency of subjects

**Fig. 1:**
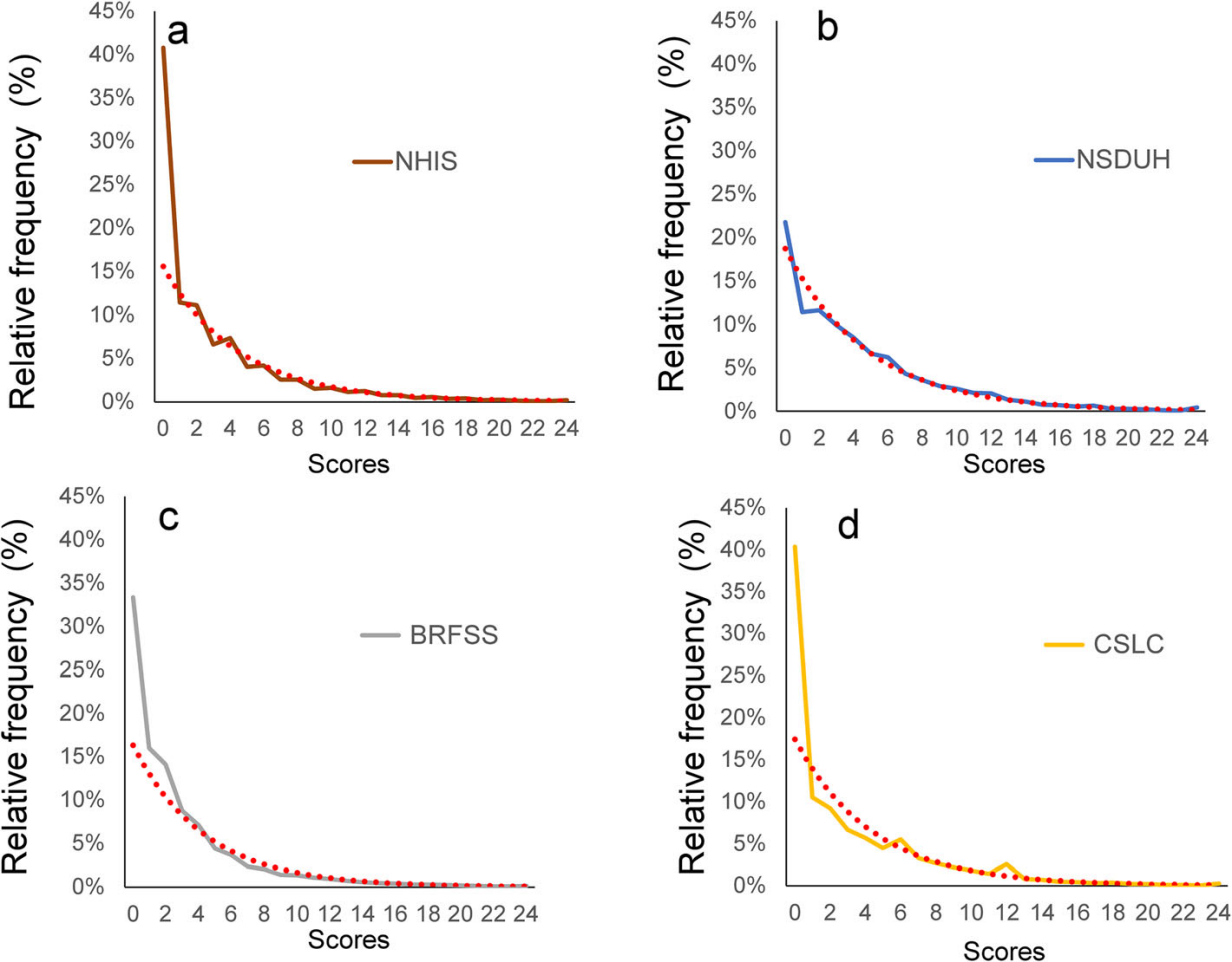
Relative frequencies of the K6 total score distributions from (**a**) NHIS, (**b**) NSDUH, (**c**) BRFSS, and (**d**) CSLC. While they were commonly right-skewed across the four surveys, the frequency of a zero score was apparently lower in the NSDUH (23.0%) than in the NHIS (40.8%), BRFSS (33.3%), and CSLC (40.2%). Red dotted lines indicate exponential regression curves. Except for at the lower end of the distributions, all exponential regression curves and the K6 distributions almost overlap. At the lower end of the distributions, the exponential regression curves deviate from the K6 distributions to some extent

Figure 1 depicts the K6 distribution (relative frequency) for the NHIS (a), NSDUH (b), BRFSS (c), and CSLC (d) (please see Supplementary Table 1 for specific numerical values). While they were commonly right-skewed across the four surveys, the frequency of zero score was apparently lower in the NSDUH (23.0%) than in those of the NHIS (40.8%), BRFSS (33.3%), and CSLC (40.2%). Red dotted lines indicate exponential regression curves for the K6 distributions. It is noteworthy that all exponential regression curves and the K6 distributions almost overlap, except for at the lower end of the distributions (Fig. 1), where the exponential regression curves deviated from the K6 distributions to some extent (see Supplementary Fig. 1 and 2 for the fitting curves of quadratic or power law models).

**Fig. 2:**
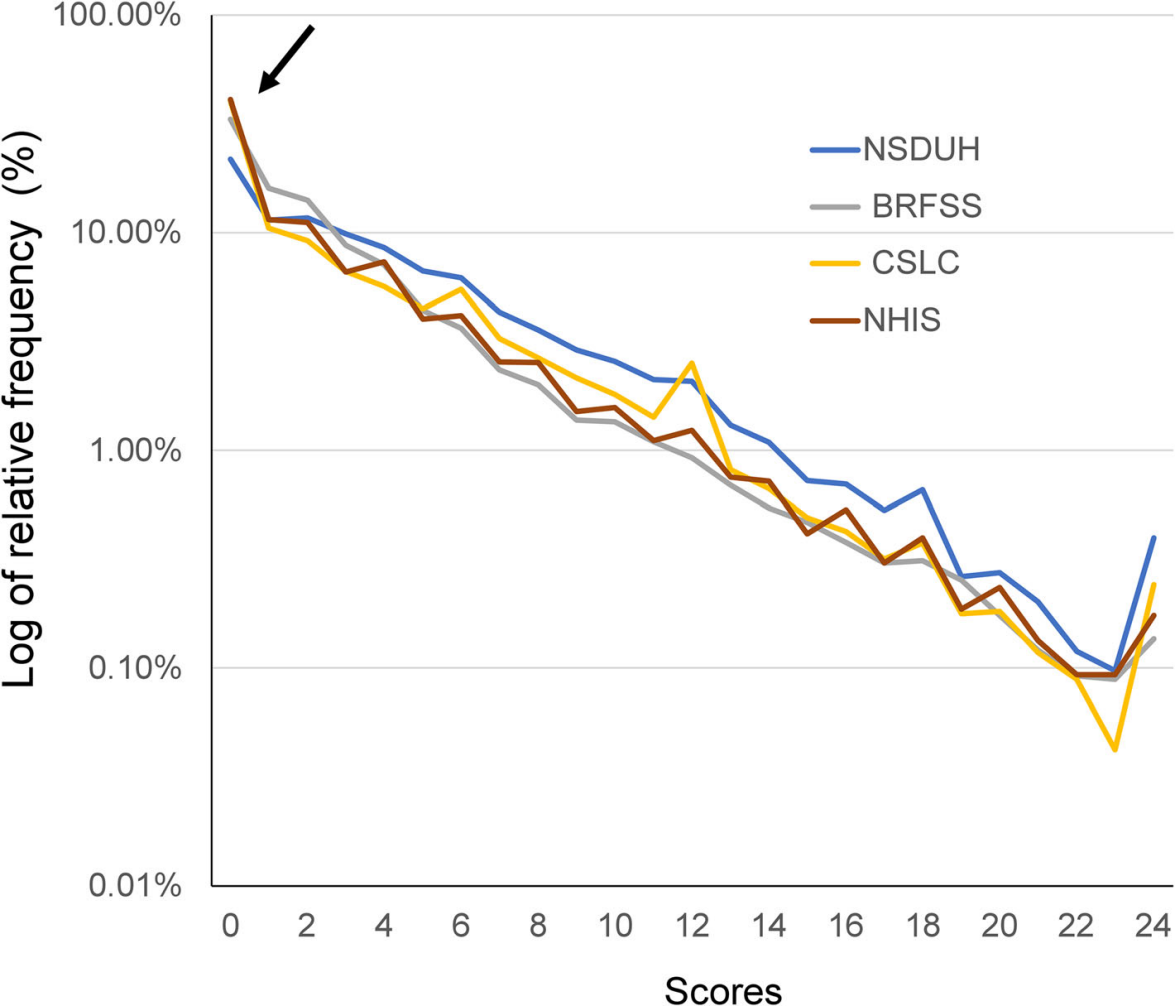
Distributions of the K6 total scores from NHIS, NSDUH, BRFSS, and CSLC on a logarithmic scale. All the K6 distributions showed a linear pattern in parallel, except at their lower end. Specifically, K6 distributions for the NHIS, BRFSS, and CSLC deviate upward from the exponential pattern at the score of 0 and at 24 points

To confirm whether all the K6 distributions approximated an exponential pattern with a similar rate parameter, they were plotted together on a logarithmic scale (Fig. 2). All the K6 distributions showed a linear pattern in parallel, except at the lower end of the distributions. Although some fluctuations were observed at the upper end of the distribution in four surveys, and at a score of 12 points in the CSLC, the slopes of all the K6 distributions were similar, consistent with the finding that the parameters of exponential regression curves for all the K6 distributions were similar (Table 3).

On the other hand, as indicated by the black arrow in Fig. 2, all the K6 distributions deviated from the linear pattern at the lower end of the distributions, consistent with the findings in a previous study [12]. Specifically, the K6 distributions for the NHIS, BRFSS, and CSLC deviated upward from the exponential pattern at the score of zero points. They also deviated upward from the exponential pattern at the score of 24 points.

## Discussion

To our knowledge, the present study is the first to compare the K6 distribution pattern among four national surveys in the United States and Japan. The K6 score distributions from the four surveys approximated an exponential distribution with a similar rate parameter except at the lower end of the distribution. These findings increase the possibility that, regardless of the different sample nations and survey methods, the K6 total distribution follows a common mathematical pattern in a general population.

The distributions of age, gender, and ethnicity differed among the four national surveys. These factors are known predictors of the prevalence of depression and anxiety in the general population [25]. However, our results showed that, regardless of the different demographics of age, gender, and ethnicity, the K6 scores exhibited a common mathematical distribution in the general population.

As mentioned in the Introduction section, previous studies have shown that, regardless of the different chosen items, the distributions of summed item scores with the same number of chosen items on the K6 and CES-D exhibit an exponential pattern with a similar rate parameter in the general population [12, 13]. The present and previous findings will help us establish a mathematical model for the distribution on similar rating scales.

It is noteworthy that a spike was observed at the upper end of the distribution in four surveys on a logarithmic scale (Fig. 2). This finding may be due to a ceiling effect, which is observed when variance in an independent variable is not measurable above the highest score. In addition, a spike at a score of 12 points was observed in the CSLC on a logarithmic scale (Fig. 2). This could be explained by the fact that Japanese people tend to choose the response option in the middle when responding to survey questionnaires [26]. In the case of the K6, a mid-point response score is 2 points; thus, given that respondents tend to choose the mid-point, a spike at a score of 12 points will occur.

Corresponding to the present results, previous studies have also demonstrated that total scores on other depression screening rating scales, such as the CES-D, PHQ-9, and CIS-R exhibit an exponential pattern, except at the lower end of the distribution in the general population [8–10]. It is unknown why total score distributions on such scales follow an exponential pattern in the general population. A possible explanation for this is that total scores on such a rating scale are expected to represent the degree of a latent trait of depression that could follow an exponential distribution in a general population. To confirm this assumption, we have proposed a process model showing how such a scale provides a discrete score from a continuous latent trait of depression. Using the process model, we performed a simulation analysis and found that when the latent trait of depression was set to an exponential distribution, simulated total scores exhibited an exponential distribution except at the lower end of the distribution [27]. These findings suggest that total scores on such a rating scale reflect an individual’s latent trait of depression. Further research is necessary to clarify the mechanisms that enable this pattern of total score distributions.

Even though samples were collected in the same country, the prevalence of SPD (a score of ≧13 on K6) was apparently higher in the NSDUH (6.4%) than in the NHIS (4.0%) and BRFSS (3.6%). This can be attributed to methodological differences, such as sampling designs and data collection methods among the three surveys. For example, NSDUH uses ACASI (audio computer-assisted self-interview), which may yield higher reports of psychological distress symptoms than does the computer-assisted telephone interview (CATI) used by the NHIS and the computer-assisted personal interview (CAPI) used by BRFSS [2].

The major limitation of this study is the lack of extensive information on the total score distribution pattern in various populations at different times. While this study compared the K6 distribution pattern across four national surveys in the US and Japan, we only analyzed each survey’s data for a particular year. Extensive research needs to be undertaken to generalize the findings to different time periods, settings, and populations. It is noteworthy that, regarding the NHIS data, a previous study has reported that the distribution of K6 total scores was stable and followed an exponential pattern with a similar parameter over the past two decades [28]. In addition, although the graphical analysis has demonstrated that the distribution of K6 total scores followed an exponential pattern with a similar parameter except at the lower end of the distribution in the general population, we could not identify the mathematical pattern at this lower end. To develop a mathematical model for total score distributions on such scales, further research is needed to identify the mathematical pattern at the lower end of the distribution.

Despite these limitations, our findings reveal that total scores on the K6 follow a common mathematical distribution irrespective of sample population. As noted in the Introduction, statistical procedures that assume a normal distribution are used to analyze the depression scale data in epidemiological studies. Our findings suggest that statistical procedures assuming normality require careful consideration when analyzing such data. Generally, the evidence that intelligent scores approximate a normal distribution has contributed to our understanding of intelligence [29]. In the same way, the fact that total scores on such a scale exhibit an exponential distribution in the general population may enhance our understanding of psychological distress and depression.

## Conclusions

The findings of this study provide evidence that the total scores on the K6 follow a characteristic distribution, regardless of sample population. Given that they show an exponential distribution with a similar parameter, we conjecture that the total scores on such a rating scale represent the degree of a latent trait of depression, which could follow an exponential distribution in a general population. The findings of this study also extend our knowledge on depression screening scales and may serve as a basis for how total scores on such a scale are distributed in a general population.

## Supplementary Information

**Additional file 1: Supplementary Figure 1.** The K6 distributions and regression model fitting curves in the four surveys. Red dotted lines indicate regression curves with quadratic term.

**Additional file 2: Supplementary Figure 2.** The K6 distributions and power law model fitting curves in the four surveys. Red dotted lines indicate power law model fitting curves.

**Additional file 3.**

## Data Availability

The datasets analyzed in the present study are available in the official repository, NHIS: https://www.cdc.gov/nchs/nhis/nhis_2018_data_release.htm. BRFSS: https://www.cdc.gov/brfss/annual_data/annual_2013.html. NSDUH: https://www.datafiles.samhsa.gov/study/national-survey-drug-use-and-health-nsduh-2014-nid13618. CSLC dataset is accessible to researchers upon approval by the Ministry of Health, Labour and Welfare of Japan through relevant application procedures: https://www.mhlw.go.jp/english/database/db-hss/cslc-index.html.

## Abbreviations

ACASI: Audio computer-assisted self-interview
AICc: Corrected Akaike’s Information Criterion
BIC: Schwarz’s Bayesian Information Criterion
BRFSS: Behavioral risk factor surveillance system
CAPI: Computer-assisted personal interviews
CATI: Computer-assisted telephone interviews
CES-D: Center for Epidemiologic Studies Depression Scale
CIS-R: Revised clinical interview schedule
CSLC: Comprehensive survey of living conditions
K6: Kessler screening scale for psychological distress
NHIS: National Health Interview Survey
NSDUH: National Survey on Drug Use and Health
PHQ: Patient health questionnaire
RMSE: Root mean square error
WHO: World Health Organization
