# Supplementary figures and images for: Mathematical pattern of Kessler psychological distress distribution in the general population of the U.S. and Japan
Source: BMC Psychiatry. 2021 Apr 10;21:188. doi: 10.1186/s12888-021-03198-y (PMC8035733; doi:10.1186/s12888-021-03198-y)

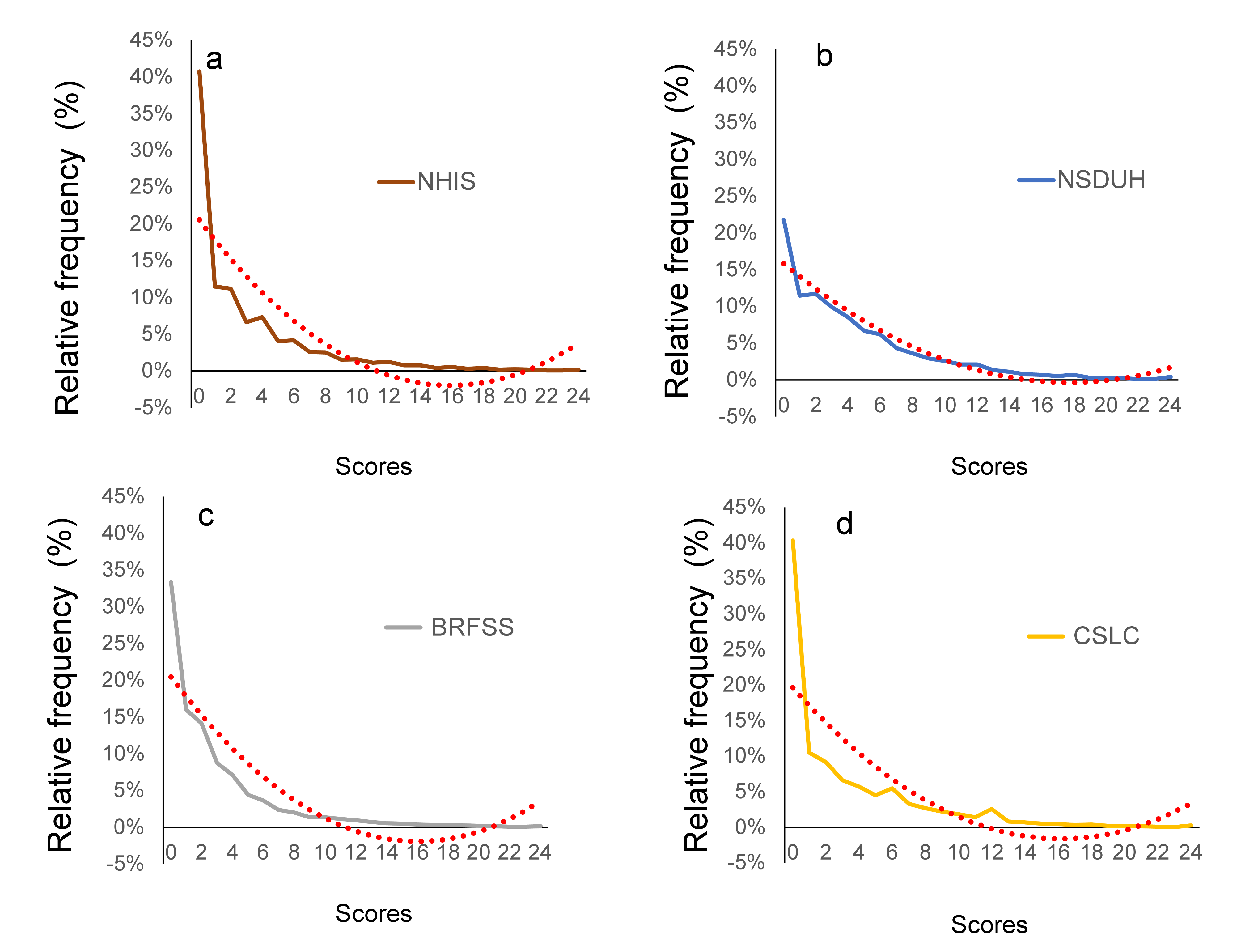

Supplement: Supplementary file 1 — Additional file 1: Supplementary Figure 1. The K6 distributions and regression model fitting curves in the four surveys. Red dotted lines indicate regression curves with quadratic term. [file 12888_2021_3198_MOESM1_ESM.tif]

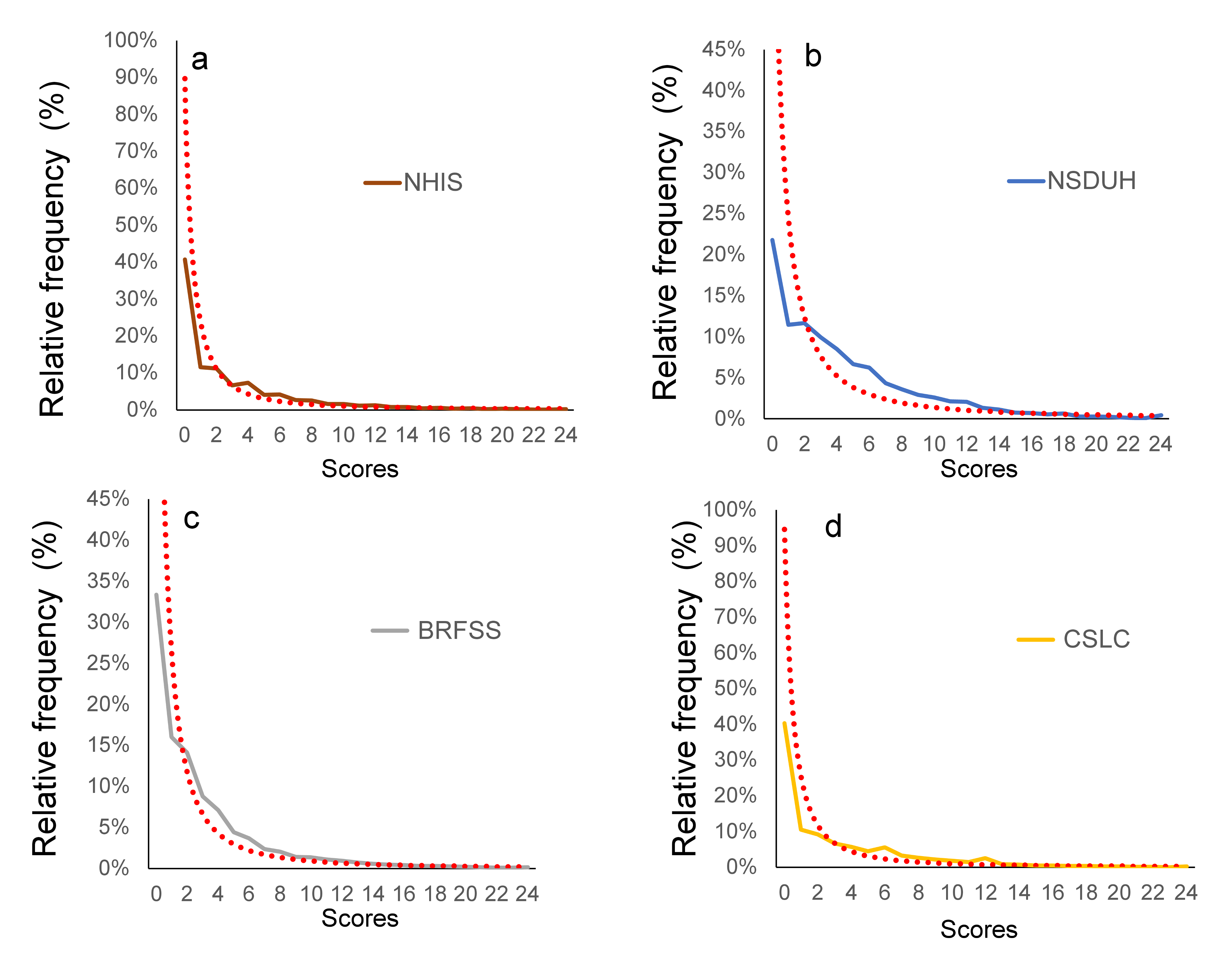

Supplement: Supplementary file 2 — Additional file 2: Supplementary Figure 2. The K6 distributions and power law model fitting curves in the four surveys. Red dotted lines indicate power law model fitting curves. [file 12888_2021_3198_MOESM2_ESM.tif]
